# Field Evaluation of a Rising Plate Meter to Estimate Herbage Mass in Austrian Pastures

**DOI:** 10.3390/s23177477

**Published:** 2023-08-28

**Authors:** Jose Maria Chapa, Barbara Pichlbauer, Martin Bobal, Christian Guse, Marc Drillich, Michael Iwersen

**Affiliations:** 1 Clinical Unit for Herd Health Management in Ruminants, University Clinic for Ruminants, Department for Farm Animals and Veterinary Public Health, University of Veterinary Medicine Vienna, 1210 Vienna, Austria; 2 Unit for Reproduction Medicine and Udder Health, Clinic for Farm Animals, Freie Universitaet Berlin, 14163 Berlin, Germany

**Keywords:** rising plate meter, herbage mass, grazing management, digitalisation, evaluation, sensor

## Abstract

Pasture management is an important topic for dairy farms with grazing systems. Herbage mass (HM) is a key measure, and estimations of HM content in pastures allow for informed decisions in pasture management. A common method of estimating the HM content in pastures requires manually collected grass samples, which are subjected to laboratory analysis to determine the dry matter (DM) content. However, in recent years, new methods have emerged that generate digital data and aim to expedite, facilitate and improve the measurement of HM. This study aimed to evaluate the accuracy of a rising plate meter (RPM) tool in a practical setting to estimate HM in Austrian pastures. With this study, we also attempted to answer whether the tool is ready for use by farmers with its default settings. This study was conducted on the teaching and research farm of the University of Veterinary Medicine in Vienna, Austria. Data were collected from May to October 2021 in five different pastures. To evaluate the accuracy of the RPM tool, grass samples were collected and dried in an oven to extract their DM and calculate the HM. The HM obtained from the grass samples was used as the gold standard for this study. In total, 3796 RPM measurements and 203 grass samples yielding 49 measurement points were used for the evaluation of the RPM tool. Despite the differences in pasture composition, the averaged HM from the RPM tool showed a strong correlation with the gold standard (R^2^ = 0.73, r_p_ = 0.86, RMSE = 517.86, CV = 33.67%). However, the results may not be good enough to justify the use of the tool, because simulations in economic studies suggest that the error of prediction should be lower than 15%. Furthermore, in some pastures, the RPM obtained poor results, indicating an additional need for pasture-specific calibrations, which complicates the use of the RPM tool.

## 1. Introduction

For dairy cattle, access to pastures has been pointed out as a welfare benefit [1] and has become increasingly demanded by consumers, especially in Europe [2]. On the one hand, meeting the nutritional requirements of cattle in pastures can be a challenge. On the other hand, grazing systems need less infrastructure to operate [1], and when correctly implemented, grazing practices can offer economic benefits for the farm [3] and reduce feed costs [4,5]. Accurate estimation of forage quantity and quality in pastures is of great interest for grazing management, as these parameters have an impact on farm economics. Hence, dairy farms that implement grazing in their feeding strategy strive for accurate estimations of the herbage mass (HM) in their pastures. This is not always easy to achieve, as to correctly estimate HM, special equipment is needed, and traditional methods, such as visual estimations, are not accurate enough and require ample experience.

Herbage mass is a measurement typically used by farmers to estimate the amount of forage and dry matter (DM) content available in pastures [4,6], and it can be defined as the kilograms of dry matter per hectare. These estimations help farmers to make decisions in grazing strategies depending on forage availability and quality [7]. Traditional methods of estimating HM are often based on visual assessments and are therefore subjective and vary from person to person. Manual cutting of grass samples followed by laboratory analysis can be more accurate but requires time, labour and special equipment that farmers may not have access to. Methods include mounting sensors to vehicles [8] and equipping drones with multispectral imaging [9], among others [10].

Precision farming (PF) technologies promise a better understanding and interpretation of data by digitalising parameters and making them available to the farmer, allowing them to make prompt and informed management decisions. With the emerging field of the digitalisation of farm processes, new methods and tools have been developed to acquire HM estimations with higher accuracies and less effort, such as rising plate meter (RPM) tools. Although these types of tools have been available for a long time, they have mainly been used to manually measure grass height in pastures. RPM tools are evolving to generate digital data and process information automatically. This could play an important role and help to digitalise HM estimations for pasture management.

In this study, we focused on the use of a semi-automated RPM tool. An RPM is a tool for measuring the compressed sward height (CSH) in pastures. Using a calibration equation, it is possible to transform CSH into HM. Previously, RPM tools were commonly used to estimate HM, and several studies have already evaluated these tools [11,12,13,14]. In another study, the output of these tools was combined with other parameters (e.g., climate data) to increase their accuracy [15]. However, these tools need calibrations for each season, pasture, grass species and geographical location due to possible differences in the performance of the tool. Therefore, the standardisation of calibration methods is needed [16]. It has been suggested that this tool may need pasture-specific calibrations, rather than regional or country-specific ones [17]. One of the downsides of these tools is that it takes time and effort to repeatably measure larger farms. However, for smaller farms, these types of tools have been shown to require less effort and time to estimate HM than other tools [9]. Hence, they may be suitable for smaller farms, such as in the case of many Austrian farms.

In this study, our aim was to evaluate whether the RPM tool is suitable for use by farmers with its default settings and to determine if it can provide meaningful results. Specifically, we evaluated the accuracy of the rising plate meter (RPM) tool in estimating HM in Austrian pastures in a practical setting.

## 2. Materials and Methods

This study was conducted at the teaching and research farm (VetFarm) of the University of Veterinary Medicine Vienna, Austria. Measurements and grass samples were taken between May and October 2021 in five different pastures with corresponding control plots (10 × 20 m) within each pasture. Plots were randomly placed inside pastures to serve as control areas in which the grass spatial composition would differ less within the plot compared to the grass spatial composition of the entire pasture. The plots were also moved between sampling dates to different parts of the pastures to avoid collecting samples in the same area.

### 2.1. Data Collection

The Grasshopper^®^ RPM tool (Grasshopper G2 sensor; True North Technologies, Shannon, County Clare, Ireland) was used to measure the CSH (in mm) in five different pastures and their corresponding plots (n = 26). This tool uses an ultrasonic sensor placed mid-way on the shaft to measure the distance from the sensor to the moveable plate and thereby determine the CSH. In addition to the RPM tool, the Grasshopper^®^ system comes with proprietary applications for tablets and smart devices. For this study, the “Grasslab” app (Grasshopper G2 sensor; True North Technologies, Ireland) was used. This app was specially designed for research purposes, whereas the standard “Grasshopper” app was designed for daily use on farms. More details about this tool can be found in McSweeney et al. [14]. The RPM tool estimates the HM of a single measurement using the formula Equation (1):

RPM HM = (CSH − 40) × 25(1)

where CSH is the height (mm) of the grass, 40 (mm) is the post-grazing height value, and 25 is the assumed DM percentage, which is set as the default. For the estimation of the HM of an entire pasture, the system averages all the CSH measurements of that pasture and uses the same formula.

To collect data using the RPM tool, approximately 75 RPM measurements per hectare were taken in a zigzag pattern when sampling pastures. For the plots, 36 RPM measurements with an approximate separation distance of 2 m were performed.

During the walk with the RPM tool, the exact positions of randomly chosen RPM measurements were marked. After completing the RPM measurements, grass samples were taken from the marked spots. In the pastures, three random spots per hectare were used to collect grass samples. Similarly, three random spots per plot were used to collect grass samples. To collect a grass sample, a 50 × 50 × 4 cm wooden frame was placed in the exact spot where an RPM measurement had been performed. Grass samples were then cut using an electric grass trimmer (Bosch ABS 10.8 LI, Robert Bosch Power Tools GmbH, Stuttgart, Germany). The samples were cut 40 mm above the ground to simulate the post-grazing or post-cutting residue and to match the default formula used by the tool. The collected grass was weighed, frozen and stored in vacuum-sealed bags for later analysis. The frozen samples were dried at 100 °C for 24 h in a drying oven (Binder BD 240, Binder GmbH, Im Mittere Ösch, Germany) to extract the DM% and calculate the HM using the formula Equation (2):

Laboratory HM = grass weight (kg) × DM% × 40,000(2)

where 40,000 represents the number of wooden frames (50 × 50 cm) fitting in a hectare. The HM calculated from the grass samples was used as the gold standard (hereafter referred to as the “laboratory method”) for the comparison to HM estimation using the Grasshopper^®^ system.

The botanical composition of all pastures was assessed by an expert at the beginning of the experiment (Table 1). Three of the pastures were used for grazing cows and two for grass cuttings.

### 2.2. Data Analysis

In total, 3865 RPM measurements and 208 grass samples were taken during the study. Of those, 69 CSH measurements outside of the measurement range of the Grasshopper^®^ system had to be excluded, which consequently led to the exclusion of five grass samples. Therefore, 3796 RPM measurements and 203 grass samples were used in the final data analysis. All data were merged and managed in Microsoft Excel (Microsoft Excel Version 2010; Microsoft Corporation, Redmond, WA, USA) spreadsheets. Statistical analyses were performed using SPSS (version 26, IBM Corp., Armonk, NY, USA). The data were analysed using Pearson correlation (r_p_), root square mean error (RMSE), the coefficient of variation (CV), mean bias (MB) and linear regression analysis. Quadratic and cubic regressions analysis was used for the comparison of results between studies and to explore their potential to estimate HM. The normality of the data was assessed using the Shapiro–Wilk test. A nonparametric Wilcoxon signed-rank test was used to evaluate the difference between the estimations from the RPM tool and the laboratory results. The Mann–Whitney U test was used to evaluate differences between plots and fields. The significance level was set at *p* < 0.05. To determine the performance of the tool, the data were analysed by (1) using the averaged HM of grass samples against the averaged HM from the RPM tool of every sampling event (n = 49) and (2) pairing individual HM results of grass samples with the corresponding RPM measurement (n = 203).

## 3. Results and Discussion

All pastures differed in their botanical composition (Table 1).

Table 2 summarises the number of samples taken, the size of each pasture and the regression analysis results.

**Figure 1:**
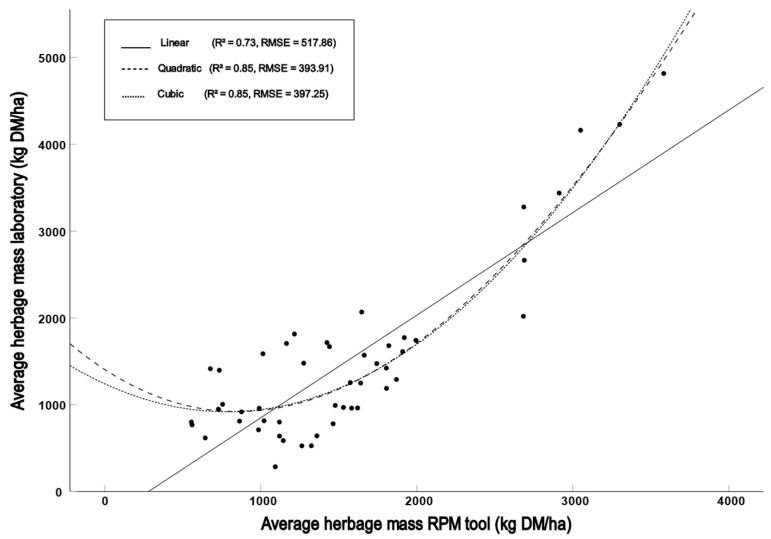
Linear, quadratic and cubic regression models of all averaged measurements taken during the study.

The best agreement of HM estimates between the laboratory and the RPM tool was observed when comparing all averaged measurements (n = 49, R^2^ = 0.734, r_p_ = 0.856, RMSE = 517.861, CV = 33.67%. Figure 1). The Wilcoxon test showed no differences (*p* = 0.293). Using quadratic and cubic regressions for estimating HM from CSH measurements obtained using the RPM tool improved the agreement with the laboratory method substantially (Table 2). This is in disagreement with Dillard et al. [13], who found only a slight improvement using the same regressions. This indicates that in some cases, using quadratic and cubic regression analysis may be better suited for HM prediction. When using the data from all five pastures combined, the RPM tool obtained acceptable results. However, the performance can vary between different pastures at the same location, which is disadvantageous in a practical setting without laboratory analysis, because otherwise, the user cannot verify the accuracy of the tool for a given pasture.

**Figure 2:**
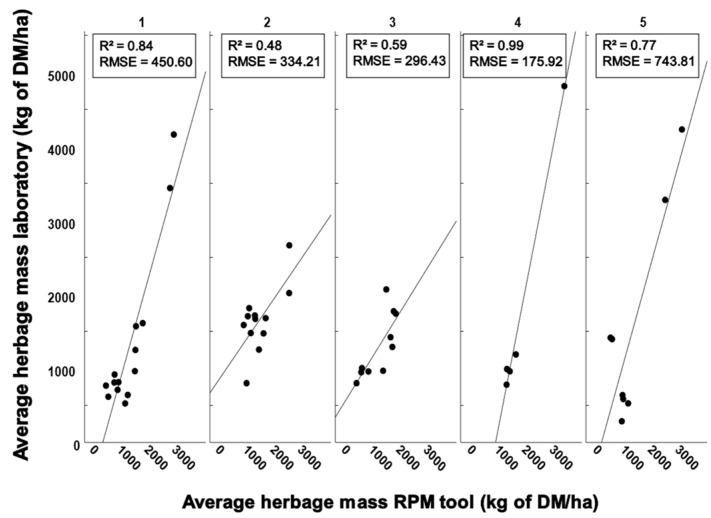
Linear regression models for individual pastures (1 to 5) examined during the study.

The agreement between the averaged HM results of individual pastures across the entire study had coefficients of determination (R^2^) of 0.845, 0.478, 0.588, 0.992 and 0.772, and there were no differences between the laboratory method and the RPM tool (*p* = 0.198, *p* = 0.754, *p* = 0.445, *p* = 0.500 and *p* = 0.779 for pastures 1 to 5, respectively, Figure 2). These different results may be attributed to the differences in botanical compositions (e.g., grass height, grass species, herbal proportion) within pastures (Table 1) or state of the pasture (e.g., grass homogeneity) [7]. Although the differences in botanical composition were not significantly different, there was a trend of worse results for pastures with a low grass content and high herbal proportion. Also, different species of grass may need special calibrations to accurately estimate HM [4].

The results obtained when comparing individual HM measurement of single grass samples directly to the corresponding RPM measurements (using the default setting of 25% DM) were R^2^ = 0.667, r_p_ = 0.817, RMSE = 643.114 and CV = 39.02% for all the measurements of this study (n = 203). Replacing the default value for the DM percentage used by the RPM tool with the actual DM % that was obtained by the laboratory analysis led to a worse outcome (R^2^ = 0.467, r_p_ = 0.683, RMSE = 814.333, CV = 44.20%). In combination with the CSH from the tool, the default value of 25% DM proved to be better suited for all pastures and seasons.

We found differences in correlations between the two methods for plots and fields. More measurements were performed for the plots (200 m^2^) (36 RPM measurements and 3 grass clippings) per m^2^ than for the fields (approx. 75 RPM measurements and 3 grass clippings per hectare). However, the higher density of measurements did not improve the performance of the tool despite the spatial botanical differences that may exist within a pasture. Using the averaged HM, the agreement of HM estimations between the laboratory and RPM tool were different for plots (R^2^ = 0.664) and fields (R^2^ = 0.819). We found differences between plots and fields (*p* = 0.0496). This shows that more measurements do not necessarily increase the accuracy of the tool, in agreement with other studies [12,18]. Other studies suggest fewer measurements per hectare (30 to 50) are sufficient for HM estimations [4,16]. Furthermore, our analysis revealed that out of 203 single measurements, 123 instances exhibited overestimations of HM, with an MB of 93 kg DM/ha. Similarly, among the averaged measurements (49 in total), 30 instances presented overestimations and an MB of 53 kg DM/ha.

In terms of the error in estimating HM, our results were similar to those of other studies [4,13,19]. Studies performing simulations of HM estimations found that to generate profits for the farm, the relative error of the HM estimations must be under 15% [5] or 10% [4]. The CV% in our study was mostly higher than the reported thresholds (Table 2), especially for the linear regression.

## 4. Conclusions

In most of the pastures, the linear regression results obtained by the RPM tool to estimate HM suggest that the tool would be of use to farmers. Replacing the linear regression for the HM estimation with either quadratic or cubic regression might improve the accuracy. However, to know if the tool is performing well on any given pasture, farmers would need to carry out additional tests, which complicates the process. The default 25% DM used in the formula of the tool to estimate HM proved to be better suited than the actual grass DM percentage. Furthermore, according to the previous literature and to justify the use of this tool economically, the errors in HM estimation must be lower than the results achieved in this study.

## Figures and Tables

**Table 1:** Botanical composition (%) and main species in pastures at the start of the study.

Pasture	Grass Proportion/Species	Legume Proportion/Species	Herbal Proportion/Species
1	75	5	20
	Oatgrass, meadow fescue, blue grass	Lucerne	-
2	70	20	10
	Meadow foxtail, oatgrass, blue grass	Lucerne	-
3	60	10	30
	Rough bluegrass	Lucerne	-
4	70	5	25
	Oatgrass, blue grass	Lucerne	-
5	60	20	20
	Meadow foxtail, orchard grass, blue grass	Lucerne	-

**Table 2:** Results of herbage mass estimations from the RPM tool compared to the laboratory results.

Analysis	r_p_	R^2^	Adjusted R^2^	RMSE (kg DM/ha)	CV%
Single measurements (n = 203)					
Linear	0.817	0.668	0.666	643.115	39.02
Quadratic	0.852	0.725	0.722	586.253	35.57
Cubic	0.851	0.725	0.721	587.676	35.66
^1^ Average measurements (n = 49)					
Linear	0.856	0.734	0.728	517.861	33.67
Quadratic	0.921	0.849	0.843	393.314	25.57
Cubic	0.922	0.850	0.840	397.252	25.83
^1^ Field (n = 23)					
Linear	0.905	0.819	0.811	461.398	29.93
Quadratic	0.938	0.881	0.869	383.789	24.89
Cubic	0.938	0.881	0.862	393.383	25.51
^1^ Plots (n = 26)					
Linear	0.814	0.664	0.650	552.886	36.03
Quadratic	0.920	0.846	0.833	381.790	24.88
Cubic	0.920	0.847	0.826	389.537	25.38
^1^ Pasture 1 (1.9 ha) (n = 14)					
Linear	0.919	0.845	0.832	450.605	30.95
Quadratic	0.984	0.968	0.962	212.161	14.57
Cubic	0.984	0.968	0.959	221.473	15.21
^1^ Pasture 2 (2.82 ha) (n = 12)					
Linear	0.691	0.478	0.426	334.216	20.92
Quadratic	0.726	0.528	0.423	335.001	20.96
Cubic	0.729	0.532	0.428	333.638	20.88
^1^ Pasture 3 (1.57 ha) (n = 10)					
Linear	0.767	0.588	0.537	296.438	21.47
Quadratic	0.769	0.592	0.476	315.367	22.85
Cubic	0.769	0.592	0.389	340.575	24.67
^1^ Pasture 4 (6.12 ha) (n = 5)					
Linear	0.996	0.992	0.989	175.929	0.08
Quadratic	0.999	0.998	0.996	100.708	0.05
Cubic	0.999	0.998	0.996	100.544	0.05
^1^ Pasture 5 (1.35 ha) (n = 8)					
Linear	0.879	0.772	0.734	743.810	49.24
Quadratic	0.959	0.921	0.889	480.377	39.80
Cubic	0.996	0.992	0.987	163.752	10.84

^1^, average results; r_p_, Pearson correlation: R^2^, R squared; RMSE, root mean square error; CV, coefficient of variation.

## Data Availability

Data are available at request.
